# Otorhinolaryngological dysfunctions induced by chronic kidney disease in pre- and post-transplant stages

**DOI:** 10.1007/s00405-020-05925-9

**Published:** 2020-03-28

**Authors:** Joanna Krajewska (Wojciechowska), Wojciech Krajewski, Tomasz Zatoński

**Affiliations:** 1 Department and Clinic of Otolaryngology, Head and Neck Surgery, Medical University in Wroclaw, Borowska 213 Street, 50556 Wroclaw, Poland; 2 Department and Clinic of Urology and Urological Oncology, Medical University in Wroclaw, Borowska 213, 50556 Wroclaw, Poland

**Keywords:** Otorhinolaryngological dysfunctions, Chronic kidney disease, Kidney transplantation, Head and neck cancer, Immunosuppression

## Abstract

**Purpose:**

Otorhinolaryngological abnormalities are common complications of chronic kidney disease (CKD) and its treatment. The main aim of this study was to provide a brief and precise review of the current knowledge regarding CKD and its treatment-related influence on head and neck organs.

**Methods:**

The Medline and Web of Science databases were searched using the terms “chronic kidney disease”, “kidney transplantation”, “immunosuppression”, “dialysis” in conjunction with “otorhinolaryngological manifestation”. Articles that did not address the topics, low-quality studies, case reports, and studies based on nonsignificant cohorts were excluded, and the full text of remaining high-quality, novel articles were examined and elaborated on.

**Results:**

Patients with CKD are prone to develop sensorineural hearing loss, tinnitus, recurrent epistaxis, opportunistic infections including oropharyngeal candidiasis or rhino-cerebral mucormycosis, taste and smell changes, phonatory and vestibular dysfunctions, deep neck infections, mucosal abnormalities, gingival hyperplasia, halitosis or xerostomia. Immunosuppressive therapy after kidney transplantation increases the risk of carcinogenesis, both related and not-related to latent viral infection. The most commonly viral-related neoplasms observed in these patients are oral and oropharyngeal cancers, whereas the majority of not-related to viral infection tumors constitute lip and thyroid cancers. CKD-related otorhinolaryngological dysfunctions are often permanent, difficult to control, have a significant negative influence on patient’s quality of life, and can be life threatening.

**Conclusion:**

Patients with CKD suffer from a number of otorhinolaryngological CKD-induced complications. The relationship between several otorhinolaryngological complications and CKD was widely explained, whereas the correlation between the rest of them and CKD remains unclear. Further studies on this subject are necessary.

## Introduction

Chronic kidney disease (CKD) is a frequent condition currently defined as reduced kidney function expressed by glomerular filtration rate (GFR) of less than 60 ml/min/1.73 m^2^ or markers of kidney damage that lasts at least 3 months irrespectively of the underlying cause [1]. The overall prevalence of CKD in United States adult population reaches 14.8%, whereas in European countries the prevalence reaches up to 15.7%, depending on the country [2].

End-stage kidney disease (ESKD) is diagnosed when patients’ GFR is less 15 ml/min/1.73 m^2^. At this stage, patients require renal replacement therapy, namely dialysis or kidney transplantation [1]. The estimated number of ESKD cases in United States reaches 661,000 [2]. Many patients with CKD require kidney transplantation and subsequent immunosuppressive treatment for the rest of their lives to prevent organ rejection [1].

As the prevalence of CKD continues to rise worldwide, the number of patients with CKD-related systemic dysfunctions, including otorhinolaryngological, will presumably increase as well [1]. CKD-induced effects of the body systems is a result of the accumulation of nitrogenous waste products, so-called “uremic toxins”, in various tissues, electrolyte imbalance, local chemical reactions due to ammonia, immunological, vascular and coagulation changes [3]. Ototoxic and immunosuppressive drugs used in CKD therapy also lead to a number of systemic complications [3]. It was established that immunosuppression in patients after kidney transplantation predisposes to various infections, especially opportunistic ones, and to malignancy occurrence [4, 5]. CKD affects a vast majority of organ systems, but the focus of this review will be on otorhinolaryngological complications of CKD both in pre- and post-transplant stage.

Some CKD-related otorhinolaryngological dysfunctions were studied more precisely than others. The most commonly analyzed abnormalities in head and neck area in patients with CKD, including renal transplant recipients (RTRs), were sensorineural hearing loss, epistaxis, candidiasis, halitosis, xerostomia, dysgeusia, lip and thyroid cancers. Additionally, in this review, the correlation between CKD and other conditions including rhinosinusitis, rhino-cerebral mucormycosis, sudden sensorineural hearing loss, deep neck infections, mucosal ulceration, lichenoid changes, oral hairy leukoplakia, tinnitus, vertigo, olfaction loss, tympanosclerosis, voice dysfunction, gingival hyperplasia, and hand and neck cancers was also reported.

### Aim of the study

The main aim of this study was to provide a brief and precise review of the current knowledge regarding CKD and its treatment-related influence on head and neck organs.

### Methods

The Medline and Web of Science databases were searched without time limit but focusing on the newest report, using the terms “chronic kidney disease”, “kidney transplantation”, “immunosuppression”, “immunosuppressive agents”, “dialysis” in conjunction with “otorhinolaryngological manifestation”, “ear”, “nose”, “throat’’, “oral cavity”, “pharynx”, “larynx”, “hearing”, “vertigo”, “head and neck cancer’’, “olfaction”, “voice”, “infection”, “sinusitis”, “tinnitus”, “tympanosclerosis”, “myringosclerosis”, “halitosis”, “epistaxis”, “candidiasis”, “xerostomia”, “taste”, and “deep neck infections”. Conditions leading to chronic kidney disease, e.g., hypertension, diabetic mellitus or connective tissue diseases were not discussed in this study. Boolean operators (NOT, AND, OR) were also used in succession to narrow and broaden the search. Auto alerts in Medline were also considered, and the reference lists of original articles and review articles were searched for further eligible sources. The search was limited to the English, German and Polish publications. Articles that did not address the topics, low-quality studies, case reports, and studies based on nonsignificant cohorts were excluded, and the full text of the remaining high-quality articles were examined and elaborated on.

## Otorhinolaryngological changes in patients with CKD (Fig. 1, Table 1)

### Hearing dysfunctions

#### Sensorineural hearing loss (SNHL)

Sensorineural hearing loss (SNHL) is a common otorhinolaryngological manifestation in patients with CKD [6, 7]. CKD is believed to be an important independent risk factor for SNHL [6, 7]. SNHL is usually bilateral in patients with CKD, and is more frequently observed in these individuals than in general the population [6, 7]. The prevalence of SNHL in CKD patients ranges from 28 to 77% [7, 8]. It was mainly diagnosed in long-lasting CKD patients and deteriorated over time [7, 8]. It was reported that the highest prevalence of SNHL occurred in individuals with estimated glomerular filtration rate (eGFR) above 45 ml/min/1.73 m^2^ [9].

**Fig. 1:**
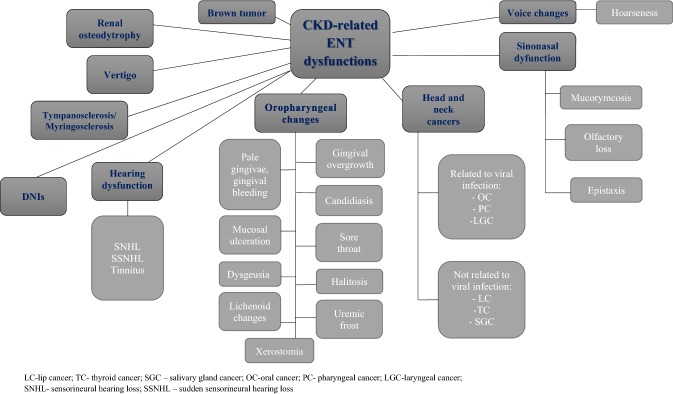
Ear, nose and throat (ENT) dysfunctions potentially related to chronic kidney disease (CKD). *LC* lip cancer, *TC* thyroid cancer, *SGC* salivary gland cancer, *OC* oral cancer, *PC* pharyngeal cancer, *LGC* laryngeal cancer, *SNHL* sensorineural hearing loss, *SSNHL* sudden sensorineural hearing loss

**Table 1:** Summary of prevalence, pathophysiology and characteristic features of otorhinolaryngological disorders in chronic kidney disease

Group of disorders	Disorder	Prevalence		Pathophysiology	Additional information
Hearing dysfunction	SNHL	28–77% of patients with CKD are affected [7, 8]		It might result from several structural and functional similarities in kidney and in inner ear, as well as similar antigenicity [8] Potential mechanisms leading to SNHL in CKD Electrolyte disturbances [6] Elevated serum urea and creatinine levels [6] Treatment (ototoxic drugs: aminoglycosides and furosemide, HD itself and prolonged treatment duration) [6, 8] Coexisting hypertension or DM [6] Vitamin D deficiency and reduction of Na+ K+ activated ATPase [8] Endolymphatic edema [8] Uremia-induced dysfunctions in nervous system, called “uremic neuropathy” leading to auditory nerve and hearing pathway alterations [8] Formation of amyloid collections in the cochlea induced by permanent HD [8] Toxic influence of aluminum on inner ear [8]	SNHL in CKD is usually bilateral and mainly affects high frequencies [8] Speech discrimination seems not to be affected in these patients [8] DPOAE are reduced or absent, and might detect subclinical cochlear dysfunction in CKD [10] ABR tests might reveal lower neural auditory conduction defined by prolongation of ABR waves [11] ABR test might be improved after HD sessions (potentially because of Ca++ ions and urea changes after HD), nevertheless hearing never return to normal [11]
	SSNHL	1.57-times higher than in GP [12]		Mechanism is unknown	Worse prognosis of recovery than in non-CKD patients while undergoing systemic steroid therapy for SSNHL [12] Promising treatment results after intratympanic steroid injections in these patients [12]
	Tinnitus	3.02-times higher in CKD than in GP [9] 4.586-higher in CKD on HD than in GP [9]		Mechanism is unclear Might be an effect of downregulation of intracortical suppression that is linked to the cochlear damage [9]	Commonly coexisting with SNHL
Oropharyngeal changes	Xerostomia	28.2–91% of patients with CKD are affected (higher with coexisting DM) [19, 20, 24]		Results from dehydration, reduced saliva flow and urea-induced changes in salivary gland morphology (fibrosis and atrophy) [21]	In patients with CKD saliva flow is 20–55% reduced [20] Cases with no measurable saliva flow are also present in these patients [20]
	Dysgeusia	43–90% of patients with CKD are affected [19, 25]		Exact mechanism is unknown It might emerge from the influence of uremic toxins on both, the central nervous system and on the teste receptors located in the peripheral nervous system [17]. High levels of urea, dimethyl and trimethylamine in saliva, reduced saliva production, altered saliva composition, reduced number of taste buds, metabolic disorders, and drugs used in treatment (mainly antihypertensive agents) play a role [17]	Commonly accompanied with “metallic taste”; sour and sweet tastes might be more significantly affected than salty and bitter tastes [17]
	Halitosis	91% of patients with CKD not on HD; 90% of patients with CKD on HD are affected [25]		Results from high urea levels (above 55 mg/dl) Alkaline nature of urea and ammonia maintain increased pH levels of saliva promoting bacteria development and unpleasant odor from oral cavity [23]	Increased risk of dental calculus formation and reduced risk of caries because of alkaline saliva pH [23]
	Sore throat	Higher than in GP *[17]		Might be a consequence of reduced saliva production, dehydration and urea decomposing commensal bacteria [17]	
	Mucosal ulceration	8.6% of patients with ESKD 1.3% of RTRs [16]		Might be a consequence of reduced saliva production, dehydration and urea decomposing commensal bacteria [17]	
	Gingival overgrowth	85% of RTRs are affected [23]		It mainly results from drug-induced changes in gingival fibroblasts and lamina propria that lead to formation of deposits of the intercellular matrix and increase in vascularity [26]	In patients in pre-dialysis or HD stage of CKD it is mainly induced by calcium channel blockers, while in RTRs by cyclosporine [26]
	Lichenoid changes/leukoplakia	8–11% of RTRs are affected [27]		Mechanism is unknown Might be a result of drug-induced reactivation of EBV in the oral epithelium [28] Negative EBV cases were also presented [23]	Usually present as painless, irregular white patches that could not be scraped of, mainly located on lateral or dorsolateral tongue and buccal mucosa [26] Frequently observed in those on cyclosporine therapy [26] No potential to malignant transformation revealed [26]
	Candidiasis	37% of patients with CKD are affects [28]		Occur because of alkaline pH that leads to modification in commensal bacteria flora [28]	Typical presentation [28] White plaques located on buccal mucosa, palate, tongue, gingivae and throat Painful and burning sensation in the oral cavity and throat Altered taste
	“Uremic frost”	Higher than in GP* [17]		Results from urea crystals depositions on oral mucosa due to saliva evaporation [17]	
	Gingival bleeding/ pallor	Higher than in GP* [26]		Occur because of anemia, platelet dysfunction induced by bacterial toxins, and anticoagulant therapy [26]	
	Oropharyngeal changes in general	97% of patients with CKD [17] according to Oyetola et al. (*n* = 90) 100% of patients with CKD [19] according to Patil et al. (*n* = 100)			
Head and neck malignancy	Lip cancer	Prevalence	Total no of studied patients	Oncogenesis induced by cyclosporine A therapy in immunocompromised RTRs [31, 39] Smoking and solar UV radiation promote oncogenesis in these patients [31]	Potentially not related to viral infection [36] LC constitutes 5–22.9% of all tumors in RTRs [23, 31] Risk of LC is higher in RTRs than in patients with CKD on HD [37] Majority of cases are invasive SSCs and are located on the vermilion of the lower lip [31, 33, 39]
		15-times higher in RTRs Laprise et al [33] 46- times higher in RTRs than in GP Krynitz et al. [40] 9.4-times increased risk in RTRs Piselli et al. [38] 47.08- times increased risk in RTRs Vajdic et al. [42]	*n* = 261,500 *n* = 10,476 *n* = 7217 *n* = 28,855		
	Thyroid cancer	Prevalence	Total no of studied patients	Might result from [41] Metabolic changes induced by chronic kidney failure, mainly hypocalcaemia-induced secondary hyperparathyroidism Decreased serum levels of selenium	Potentially not related to viral infection [36] Risk of TC is elevated both, after kidney transplantation and in patients with CKD on HD; risk is higher in ESKD than in RTRs [41, 42]
		6.77-times increased risk in RTRs van Leeuwen et al. [39] 1.85-times increased risk in organ recipients Mowery et al. [5] 6.9-times increased risk in RTRs Vajdic et al. [42]	*n* = 8173 *n* = 19 173 *n* = 28,855		
	Salivary gland cancer	Prevalence	Total no of studied patients	Mechanism is unknown Potentially results from lack of immunologic surveillance after organ transplantation [5, 38]	Potentially not related to viral infection [36] Risk seems to be increased in RTRs [5, 38] Studies on the risk in pre-transplantation CKD are lacking
		2.91-times increased risk after organ transplantation Mowery et al. [5] 5.8- times increased risk in RTRs Piselli et al. [38]	*n* = 19,173 *n* = 7217		
	Oral cancer	Prevalence	Total no of studied patients	Lack of immunosurveillance in RTRs because of drug-induced immunosuppression. Immunosuppression affects tumor immunosurveillance and reduces immunologic control of oncogenic viral infection subsequently leading to cancer development [30] Oncogenic viruses involved in oral carcinogenesis—especially HPV [32, 34–36] Smoking and alcohol consumption as coexisting promoting factors [34]	RTRs are at higher risk of carcinogenesis than those at pre-transplant stage of CKD [30]
		3.2-times increased in organ recipients Grulich et al. [36] 4-times increased in RTRs Makitie et al. [34] 2.42-times increased risk in CKD patients on HD Taborelli et al. [37]	*n* = 31,977 *n* = 2890 *n* = 3407		
	Pharyngeal cancer	Prevalence	Total no of studied patients	Lack of immunosurveillance in RTRs because of drug-induced immunosuppression Immunosuppression affects tumor immunosurveillance and reduces immunologic control of oncogenic viral infection subsequently leading to cancer development [30] Oncogenesis induced by oncogenic viruses, especially HPV (oro- and hypopharyngeal cancers), and EBV (nasopharyngeal cancer) [32, 34–36] Smoking and alcohol consumption as coexisting promoting factors [34]	RTRs are at higher risk of carcinogenesis than those at pre-transplant stage of CKD [30]
		3.2-times increased in organ recipients Grulich et al. [36] 4-times increased in RTRs Makitie et al. [34]	*n* = 3977 *n* = 2890		
	Laryngeal cancer	Prevalence	Total no of studied patients	Lack of immunosurveillance in RTRs because of drug-induced immunosuppression. Immunosuppression affects tumor immunosurveillance and reduces immunologic control of oncogenic viral infection subsequently leading to cancer development [30] Oncogenic viruses involved in laryngeal carcinogenesis—especially HPV [32, 34–36] -Smoking and alcohol consumption as coexisting promoting factors [34]	RTRs are at higher risk of carcinogenesis than those at pre-transplant stage of CKD [30]
		4-times increased in RTRs Makitie et al. [34] 2.03-times increased risk in CKD patients on HD Taborelli et al. [37]	*n* = 2890 *n* = 3407		
	HNC in general				80% (n = 2284) of all HNC in RTRs are of cutaneous type [34] Makitie et al 93% (n = 359) of post-transplantation HNC are of cutaneous type [43] Rabinovisc et al
Sinonasal disorders	Epistaxis	Higher than in GP* [3]		Predisposing mechanism Collection of toxins (mainly high blood urea levels) not properly removed by kidneys [3] Anemia and coagulation dysfunctions [3] Bacteria colonizing nasal cavity that decompose urea to ammonia [3]	One of the most common sites of bleeding in patients with uremia [3] Nasal bleeding could be resolved/reduced after blood urea level is normalized [3]
	Mucormycosis (rhino-cerebral)	52–56.25% of RTRs are affected [4]		Might result from [4] Cytotoxic drugs and steroids incorporation Prolonged antibiotic therapy Drug-induced granulocytopenia Uremia Hyperglycemia Poor nutritional status	Sinonasal/rhino-cerebral mucormycosis mainly presents as headache, facial swelling and pain (especially over affected areas), nasal discharge, and necrotic lesions on the face, nasal cavities, or palates [4, 45] Rhino-cerebral form is most common form of mucormycosis in RTRs [4] Maxillary and ethmoid sinuses are mainly affected [45]
	Olfactory loss/dysfunction	56% of patients with ESKD are affected [48]		Exact mechanism is unknown It might result from uremia-induced negative effect on peripheral nerve conduction and central cognitive functions [47]	Might be reversible- improvement of proper olfaction observed after renal transplantation and after dialysis session [47] Olfactory identification and discrimination are mainly affected; thresholds seem to remain unchanged [47]
	Rhinosinusitis	Consensus is lacking No increase in RTRs [44] according to Ryu et al.		Might result from decreased immunologic response especially after organ transplantation [45, 46]	Sinonasal examination is not recommended in asymptomatic individuals because of no exacerbations observed in RTRs [44] CT of paranasal sinuses before organ transplant not recommended in asymptomatic individuals because of the high rate of false positive results [44] Relatively low incidence of rhinosinusitis in RTRs might result from persistent low-dose prednisone therapy withholding the inflammatory responses that frequently promote CRS [44]
Voice dysfunction	Hoarseness	24–60% of patients with ESKD are affected [50]		Potential mechanism [15] Excessive fluid and toxins accumulation, and acid–base imbalance Vocal cord edema Decreased pulmonary function Abnormal coordination between central nervous system and peripheral phonatory structures Laryngeal muscles fatigue	Patients with ESKD on HD might suffer from temporary post-dialysis hoarseness as a result of HD-induced dehydration, reduction of the vocal cord size and increase in subglottic pressure [50]
Bony changes	Renal osteodystrophy in H&N area	Higher than in GP* [16]		Bone metabolic changes induced by chronic renal insufficiency [16, 26] Phosphate retention and reduced vitamin D conversion result in hypocalcaemia and subsequent production of parathormone (PTH) that stimulates bone resorption [16, 26]	May present as demineralization of the mandible and maxilla, loss of the lamina dura, and metastatic calcification in hard tissues [26] The most common abnormalities are temporomandibular joint deformation, maxillofacial fractures and malocclusion [26]
	Brown tumor	1.5–1.7% of patients with CKD-induced secondary parathyroidism are affected [29]		Occurs secondary to CKD-induced hyperparathyroidism [29]	Mainly observed in mandible, palate or facial bones; less frequently in skull bones and paranasal sinuses [29]
Middle ear dysfunction	Tympano-sclerosis/myringo-sclerosis	Consensus is lacking Potentially higher in CKD on HD than in GP [13]		The accumulation of serum phosphate binding to free calcium may lead to calcification in middle ear structures [13]	Increased risk of myringosclerosis was found in CKD patients on HD lasting longer than 3 years [13] No similar association was found between HD duration and myringosclerosis formation [14]
Vestibular dysfunction	Vertigo	Higher than in GP* [15]		Mechanism is unclear Might be an effect of toxic products retention with subsequent vasculopathy, vestibulocochlear neuropathy and vascular calcification in the labyrinth [10]	Abnormal responses in oculomotor and combined vestibular-evoked myogenic potential (VEMP) tests in patients with CKD [10] Negative correlation between eGFR and labyrinth function [15]
Neck disorder	Deep neck infections	Higher than in GP*, especially in patients on HD [51, 54–56] 3-times higher risk of serious infection in CKD on HD than in GP [54] Need for hospitalization because of serious DNI is almost 10-times higher in CKD on HD than in GP [54]		Predisposing mechanisms Uremia (interfering with primary host defense mechanisms subsequently elevating the risk of bacterial infections) [51, 54] Neutrophil dysfunction induced by impaired glucose metabolism, secondary hyperparathyroidism, iron accumulation, malnutrition and HD [54] Constant immunosuppression and immunity alterations that favor the growth of opportunistic organisms in RTRs [56]	Dysfunctional neutrophils present malfunctioning chemotaxis, degranulation and phagocytosis, subsequently failing to prevent CKD host from developing infection [54] CKD constituted 3rd most common condition predisposing to DNIs following DM and nasopharyngeal cancer after radiotherapy [58]

*HD* hemodialysis, *GP* general population, *DM* diabetes mellitus, *ESKD* end-stage kidney disease, *RTRs* renal transplant recipients, *eGFR* estimated glomerular filtration rate, *CT* computed tomography, *CRS* chronic rhinosinusitis, *SCC* squamous cell carcinoma, *TC* thyroid cancer, *LC* lip cancer, *H&N* head and neck

^*^Not precisely estimated value

The high number of patients with CKD suffering from SNHL might result from several structural and functional similarities in kidney and in inner ear [8]. The most important similarity is the active transportation of electrolytes and fluids carried out in the glomerular basement membrane and in the cochlear stria vascularis [8]. It is a result of the presence of Na + K + ATPase pump and a carbonic anhydrase enzyme [8]. Additionally, it was also found that the cochlea and kidney share similar antigenicity [8]. To support that, there are some diseases and syndromes (e.g., Alport syndrome) that affect both, inner ear and kidney.

It was suggested that SNHL in patients with CKD could result from electrolyte disturbances, elevated serum urea and creatinine levels, treatment (ototoxic drugs, hemodialysis itself and prolonged treatment duration), hypertension or commonly coexisting DM [6]. The most widely discussed ototoxic drugs used in managing CKD are aminoglycosides and furosemide [6]. Vitamin D deficiency and reduction of Na+ K+ -activated ATPase were also implicated in SNHL [8]. It was suggested that inhibition of Na+ K+ -activated ATPase that is crucial in providing proper ionic gradient in the inner ear, could be the main cause of sensorineural hearing dysfunction in uremic patients [8]. Another dysfunction predisposing to SNHL in patients with CKD is endolymphatic edema [8]. It was previously described that endolymphatic hydrops was related to low-frequency SNHL and could explain hearing amelioration after hemodialysis [8].

Uremia-induced dysfunctions in nervous system, called “uremic neuropathy”, could also lead to auditory nerve and hearing pathway alterations [8]. This observation was supported by Auditory Brainstem Response (ABR) test conducted in patients with CKD by various authors [7]. It was observed that cases of SNHL in patients with CKD resulted more commonly from cochlear dysfunction than from retrocochlear hearing pathology [8].

The formation of amyloid collections in the cochlea induced by permanent hemodialysis might also lead to hearing dysfunction [8]. Finally, hearing loss might result from toxic influence of aluminum on inner ear in these patients [8]. In addition to that, it was reported that duration of hemodialysis constituted the only independent predictor of SNHL [6].

SNHL should be confirmed using audiological tests. The most common audiometric abnormality observed in patients with CKD was high-frequency loss and a notch at 6 kHz [8]. Speech discrimination seemed not to be affected in these patients [8]. Distortion product otoacoustic emissions (DPOAEs) are evoked responses produced when the cochlea is stimulated simultaneously by two pure tones [10]. DPOAEs testing is sensitive to detect cochlear dysfunction, even the subclinical one [10]. DPOAEs were absent in a significant number of patients with CKD in various studies [10]. Auditory Brainstem Response (ABR) is an objective non-invasive electrophysiological test measuring the retrocochlear part of the auditory pathway, up to the brainstem level, in response to sounds [11].

Several authors presented that patients with ESKD expressed slower neural auditory conduction defined by prolongation of ABR waves [11]. It was concluded that in patients with CRD conduction times in ABR test were improved after the session of hemodialysis, nevertheless hearing never returned to normal [11]. Differences in ABR responses before and after hemodialysis might have resulted from various calcium ions (Ca++) levels [11]. According to that, it was suggested that hearing loss might have inversely correlated with the amount of hemodialysis sessions [6].

#### Sudden sensorineural hearing loss

Interestingly, studies showed that patients with CKD were 1.57-times more prone to develop sudden sensorineural hearing loss (SSNHL) than the general population [12]. The risk was even higher in patients with CKD and coexisting DM [12]. Whereas the potential etiologic factors of SNHL were indicated, the etiology of SSNHL in this population remains unclear [12]. Kang et al. presented that patients with CKD and coexisting SSNHL expressed worse recovery prognosis than non-CKD individuals when treated with systemic glucocorticosteroids, a first-line treatment for SSNHL [12]. In contrast to that, promising results in SSNHL treatment in patients with CKD were accomplished by intratympanic steroid injections [12].

#### Tinnitus

Tinnitus is a perception of the sound in the absence of auditory stimulus from the outside, and is mainly a result of auditory pathology [9]. It might be a coexisting symptom in SNHL [7]. Tinnitus is an effect of downregulation of intracortical suppression that is linked to the cochlear damage, nevertheless the exact mechanisms leading to tinnitus in patients with CKD remain unclear [9]. A population-based study on a large cohort conducted by Shin et al. revealed that CKD is an important and independent risk factor for tinnitus [9]. The authors found that patients with CKD were 3.02-times more prone to develop tinnitus than general population, especially those with severe renal dysfunction [9]. The risk was higher in females aged less than 30 years, and reached 4.586-increase in patients on hemodialysis [9]. Tinnitus was also observed as a common accompanying symptom in patients with SSNHL on hemodialysis [12].

### Tympanosclerosis, myringosclerosis

Abnormal kidney function, defined by abnormal eGFR, in patients with CKD lead to serum phosphate accumulation [13]. Serum phosphate has the ability to attach to free calcium-producing precipitates and induce subsequent calcification [13]. Decreased amount of free calcium in serum stimulates parathyroid gland to produce parathormone (PTH) [13]. Calcification was observed in patients with CKD mainly in arteries and viscera [13]. Calcification could also be formed in the lamina propria of the middle ear mucosa, however studies on this subject are sparse [13]. Matrix vesicles were presented as important elements of abnormal tissue calcification [14]. They also participated in other types of calcification, including tympanosclerosis [14]. It was suggested that patients with CKD were more prone to develop myringosclerosis, a type of tympanosclerosis that affects tympanic membrane [14]. Nevertheless, the correlation between the amount of serum phosphorus, calcium, magnesium or PTH, and myringosclerosis occurrence was not observed [13, 14]. According to El-Anwar et al. the increased risk of myringosclerosis was found in CKD patients on hemodialysis lasting longer than 3 years, whereas Caldas et al. study did not reveal similar association between hemodialysis duration and myringosclerosis formation [13, 14]. The exact correlation between CKD and both, tympanosclerosis and myringosclerosis development requires further analysis.

### Vestibular dysfunction

CKD-related electrolytic and osmotic alterations that affect cochlea, could have an influence on the labyrinth [10]. Patients with CKD are at increased risk of developing vestibular dysfunction in comparison with healthy population [15]. It was claimed that eGFR, a parameter expressing kidney function, negatively correlates with vestibular function [15]. Despite the fact that the exact cause of vertigo in patients with CKD remains unclear, the suggested potential etiologic factors were toxic products retention with subsequent vasculopathy, vestibulocochlear neuropathy and vascular calcification in the labyrinth [10].

Oculomotor and combined vestibular-evoked myogenic potential (VEMP) tests presented abnormal responses in patients with CKD supporting the observation of decreased vestibular function in these individuals [10].

### Oropharyngeal changes

Oropharyngeal lesions in patients with CKD are very common. Oropharyngeal diseases constitute a potential and preventable cause of poor health outcomes in patients with CKD. Poor CKD-related oropharyngeal health induces systemic inflammation in patients with CKD that accompanied by malnutrition, predisposes to cardiovascular diseases and increases mortality rate in this population [16]. The majority of oropharyngeal abnormalities in this population is a result of the increased level of urea in the saliva [16]. Urea is apportioned by urease into ammonium ions and carbon dioxide that leads to high, alkaline pH of saliva [16]. Immunosuppression, adverse effects of drugs used in therapy, electrolyte imbalance, restricted diets and malnutrition are other causes or oropharyngeal lesions in CKD [17].

Patients with CRD presented various oropharyngeal abnormalities including halitosis, xerostomia, periodontitis, dysgeusia, candidiasis, parotitis, abnormal lip pigmentation, burning mouth sensation and ulcerations [17]. Dental abnormalities also constituted a considerable amount of all oral findings in patients with CRD [18].

It was strongly suggested that oral manifestations and several salivary markers, namely pH, urea, and calcium should be assessed in patients with CRD, especially those on hemodialysis [17]. Oyetola et al. reported that 97% of patients with CKD developed oral lesions, whereas the prevalence was even higher (100%) in a study conducted by Patil et al. [17, 19].

Mansourian et al. reported that patients after renal transplantation were more prone to develop oral lesions than patients on hemodialysis [17, 19]. The most common oral lesion in the group of kidney transplantation recipients was xerostomia [17, 19]. In contrast to that, Ruokonen et al. conducted an interesting study revealing that renal transplant recipients (RTRs) presented better oral health than those in pre-dialysis stage of CKD [18]. However, burning mouth sensation, xerostomia, dysphagia, and dysgeusia were more commonly observed after kidney transplantation in this study [18]. The prevalence of oropharyngeal lesions in patients with CKD might have been modified by other coexisting diseases, with diabetes mellitus (DM) being one of the most frequent one.

#### Xerostomia

Xerostomia, a subjective sensation of dry mouth, commonly accompanied by difficulties in chewing, swallowing and tasting, is very often observed in patients with CKD [20]. It also predisposes to the development of oral infections and oral lesions [20]. Ruokonen et al. suggested that xerostomia is a symptom that most significantly affected quality of life (QoL) in patients with CKD [18]. There were several proposed mechanisms contributing to xerostomia development. The principal ones were dehydration, reduced saliva flow and changes in salivary gland morphology [21]. Therapy incorporating certain drugs in treatment protocol, namely immunosuppressive agents, opioids, corticosteroids, and antimicrobials could also predispose to hyposalivation [21].

Whereas in general population the saliva flow reaches approximately 0.3–0.5 ml/min, in patients with CKD on hemodialysis the flow is 20–55% reduced [20]. There were also cases with even no measurable saliva flow [20]. Interestingly, it was found that renal transplantation led to significant increase in saliva flow and reduced symptoms of xerostomia [21]. Additionally, salivary flow rate might even have returned to normal after renal transplantation [21]. It was suggested that decreased salivary flow rate in pre-transplantation stage of CKD resulted from the above-mentioned, urea-induced abnormalities in salivary glands, intake of several medications and limited fluid intake [21].

Salivary gland morphology in CKD presents fibrosis or atrophy. Postorino et al. conducted a histological evaluation of minor salivary glands in patients with CKD on hemodialysis revealing that 41% of the subjects presented significant atrophy of minor salivary glands [22]. Additionally, it was also reported that xerostomia predisposed to candidiasis and suppurative sialadenitis [23].

The prevalence of xerostomia in patients with CKD on hemodialysis ranged from 28.2 to 91%, according to various authors [19, 20, 24]. Such wide discrepancy might have resulted from various definitions and criteria used to diagnose xerostomia. A meta-analysis conducted by Ruospo et al. revealed that 48.4% of patients with ESKD presented xerostomia [16]. Swapna et al. found that 62% of nondiabetic patients with CKD on hemodialysis developed xerostomia in comparison to 78.7% of diabetics with CRD on hemodialysis [25]. According to that, the authors suggested that the prevalence of dry mouth in patients with CKD and coexisting DM was higher than in those with CKD alone [25].

#### Dysgeusia

Dysgeusia, commonly accompanied by metallic taste, in patients with CKD is related to high amounts of urea, dimethyl and trimethylamine in saliva, reduced saliva production, altered saliva composition, reduced number of taste buds, metabolic disorders, and drugs used in treatment (mainly antihypertensive agents) [19]. It was suggested that sour and sweet tastes might have been more significantly affected than salty and bitter tastes [17].

The exact mechanisms leading to abnormal taste perception in patients with CKD have not been elucidated yet. However, it was suggested that it could emerge from the influence of uremic toxins on both, the central nervous system and on the teste receptors located in the peripheral nervous system [17]. According to various authors the incidence of taste disturbances in patients with CKD ranged from 43 up to 90% [19, 25]. Swapna et al. found that 90% of nondiabetic patients with CKD on hemodialysis presented altered taste sensation [25]. The authors reported that the prevalence was higher than observed in diabetics with CKD on hemodialysis, in diabetics with CKD not on hemodialysis, and in nondiabetics not on dialysis (68%, 74% and 65%, respectively) [25]. Nascimento et al. found that 31.1% of patients with CKD experienced dysgeusia [24]. The majority of them presented the sensation of bitter taste (69.5%), followed by metallic taste sensation (17.4%), and abnormal sweet taste sensation (13%) [24]. The authors also revealed a significant correlation between dysgeusia and simplified oral hygiene index (OHI-S) [24].

#### Halitosis

Halitosis is an unpleasant odor from the oral cavity. It results mainly from oropharyngeal, sinonasal or dental chronic diseases, poor oral hygiene, gastrointestinal or systemic diseases [19]. In patients with CKD, halitosis is a very common condition that is mainly associated with high urea levels [23]. It was found that severe halitosis occurred when the blood urea levels reached above 55 mg/dl [23]. Alkaline nature of urea and ammonia maintain increased pH levels of saliva and bacterial biofilm, promoting dental calculus formation and reducing the risk of caries in patients with CKD [23].

In the study conducted by Swapna et al. uremic odor was observed in 91% of nondiabetic patients with CKD not on dialysis, in 90% of nondiabetics with CKD on hemodialysis, in 76% of diabetics with CRD not on hemodialysis, and 75% of diabetics with CRD on hemodialysis [25]. 53.3% of patients with CKD on hemodialysis presented halitosis in another study [23].

#### Sore throat

Sore throat is a common complaint in patients with CKD [17]. It is mainly a result of oropharyngeal dryness and ulcerations that are consequences of reduced saliva production, dehydration and urea decomposing commensal bacteria [17].

#### Mucosal ulceration

According to meta-analysis conducted by Ruospo et al., 8.6% of studied populations with ESKD on dialysis (*n* = 832), and 1.3% of RTRs (*n* = 453) presented mucosal ulcerations, respectively [16].

#### Gingival overgrowth

Gingival overgrowth in patients in pre-dialysis and hemodialysis stage of CKD was mainly induced by calcium channel blockers, while in RTRs it mainly resulted from cyclosporine use [26]. The combined therapy based on both, cyclosporine and calcium channel blocker (nifedipine) might have increased the incidence and severity of gingival overgrowth [23]. Similar, drug-induced adverse effect was not observed for tacrolimus [23]. Gingival hyperplasia resulted from cyclosporine-induced changes in gingival fibroblasts and lamina propria that led to formation of deposits of the intercellular matrix and increase in vascularity [26]. It was observed that gingival overgrowth might be found in up to 85% of RTRs [23]. According to Proctor et al. children and adolescents were more susceptible to develop this abnormality [23].

#### Other mucosal changes

Lichenoid changes and oral hairy leukoplakia are most commonly observed in patients with CKD after kidney transplantation [26]. The prevalence of these disorders in RTRs ranges between 8 and 11% [27]. It was suggested that oral lichenoid lesions (OLL) might have been a result of drug-induced reactivation of the Epstein–Barr virus (EBV) in the oral epithelium [26]. Nevertheless, EBV-negative cases of OLL were also observed in patients with CKD [23]. In individuals with CKD, OLL usually appeared as painless, irregular white patches that could not be scraped of, and were mainly located on lateral or dorsolateral tongue and buccal mucosa [26]. These changes were frequently present in patients on cyclosporine therapy [26]. Generally, OLL have no potential to malignant transformation thus, treatment is not recommended in the majority of cases [27].

Another type of white patches called “uremic frost” can be seen in patients with CKD as a result of deposition of urea crystals [17]. The majority of “uremic frost” changes were observed on patients’ skin after perspiration, nevertheless they were also found on oral mucosa due to saliva evaporation [17].

Patients with CKD may also commonly present pale gingivae and spontaneous, uninduced gingival bleeding [26], that results from anemia, platelet dysfunction induced by bacterial toxins, and is intensified by anticoagulant therapy and improper function of vascular wall cells [26].

#### Candidiasis

Patients with CKD are often affected by oropharyngeal candidiasis [17]. It was reported that candidiasis developed in up to 37% of individuals with CKD [28]. Candidiasis, the fungal infection caused mainly by *Candida albicans*, is a result of alkaline pH, leading to modification in commensal bacteria flora [28]. Oropharyngeal candidiasis typically presents as: (1) white plaques located on buccal mucosa, palate, tongue, gingivae and throat; (2) painful and burning sensation in the oral cavity and throat, and (3) altered taste [16].

According to meta-analysis conducted by Ruospo et al., oral candidiasis was detected in 22.2% of patients with 1–5th stage of CKD, in 19.6% of patients with ESKD, and in 13.3% of RTRs [16]. The frequency of oral candidiasis in patients with ESKD increased with time on dialysis [16].

### Soft tissue and bony changes

#### Renal osteodystrophy-induced changes in head and neck area

Renal osteodystrophy commonly observed in patients with CKD might lead to oral consequences, namely demineralization of the mandible and maxilla, loss of the lamina dura, and metastatic calcification in hard tissues [16]. Renal osteodystrophy is described as an alteration of bone morphology induced by CKD [26]. Chronic kidney insufficiency changes bone metabolism in various mechanisms [26]. Phosphate retention and reduced vitamin D conversion result in hypocalcaemia and subsequent production of PTH that stimulates bone resorption [26].

The most common implications of renal osteodystrophy in head and neck area comprised temporomandibular joint deformation, maxillofacial fractures and malocclusion [26].

#### Other changes

It was also suggested that CKD predisposed to soft tissues and parotid gland calcification, as well as to brown tumors development [29]. Brown tumor is a type of focal osteitis fibrosa cystica induced by secondary hyperparathyroidism [29]. The estimated prevalence of brown tumor reached 1.5–1.7% of patients with secondary hyperparathyroidism induced by CKD [29]. In otorhinolaryngological practice it was mainly observed in mandible, palate or facial bones, and less frequently in skull bones and paranasal sinuses [29].

### Malignancy in the head and neck area

Among all patients with CKD, RTRs were at higher risk of carcinogenesis than those at pre-transplant stage of CKD [30]. The most crucial factor predisposing to cancer development in RTRs is drug-induced immunosuppression [31]. The risk of carcinogenesis in organ recipients increases with duration and intensity of immunosuppressive therapy, and is inversely related to recipient age [31]. Immunosuppression affects tumor immunosurveillance and reduces immunologic control of oncogenic viral infection subsequently leading to cancer development [30].

Cancer-related mortality was higher in RTRs than in general population [32]. Moreover, the analysis conducted by the Australia and New Zealand Transplant Registry (ANZTR) revealed that cancer had exceeded cardiovascular disease as the major cause of death after organ transplant.

Cancers constitute 10–47% of deaths in RTRs and are among top causes of death in these patients [30]. The commonly observed tumors in this group are lip cancer (LC), thyroid cancer (TC), melanoma, non-melanoma skin cancer (especially squamous cell cancer), post-transplant lymphoproliferative disease (PTLD), Kaposi sarcoma, non-Hodgkin lymphoma, carcinomas of the vulva and perineum, hepatobiliary tumors, and genitourinary carcinomas [30, 32]. Increased cancer risk in RTRs was observed for various tumors that are related to persistent viral infection [30]. It was reported that RTRs were 2- to 3-times more prone to develop neoplasms, especially those related to viral infection, than general population [30, 32]. The uppermost risk in RTRs was observed for LP and non-melanoma skin cancers (15-times increased risk), for non-Hodgkin lymphoma and PTLD (8-times increased risk), and for anogenital tumors (4-times increased risk) [33].

Interestingly, according to Mäkitie et al. the risk of lip and non-melanoma skin cancers in RTRs was 35-times increased, while the risk of other head and neck cancers was 4-times increased, in comparison with general population [34]. This study analyzing the 10 years follow-up period reported that 80% of all head and neck cancers were of cutaneous type [34]. The significant increase in the prevalence of several head and neck tumors after kidney transplant, namely oral, salivary and non-melanoma skin cancer was found by Al-Qurayshi et al. [35]. Nevertheless, it was reported that in otolaryngology practice, RTRs were predisposed to develop head and neck squamous cell carcinoma (HNSSC) both related and unrelated to a latent viral infection [35]. High prevalence of carcinogenesis induced by oncogenic viruses was also observed for patients infected by human immunodeficiency virus (HIV) [36]. It most likely emerged from the lack of immunosurveillance [36]. The most important oncogenic viruses in RTRs were human papilloma virus (HPV), human herpes virus 8 (HHV-8), Epstein–Barr virus (EBV) and Merkel cell polyomavirus [32]. It was reported that the risk of HPV-related oral and pharyngeal carcinomas was 3.2-times increased in comparison with healthy population [36].

In contrast to that, organ recipients were also more prone to develop tumors not related to viral infection (e.g., lip or thyroid cancers) [36]. It was revealed in a study conducted by Grulich et al. presenting that the prevalence of these cancers was much less frequent in HIV-positive patients than in organ recipients [36]. On the other hand, the incidences of HPV-related cancers (laryngeal and oropharyngeal cancers) and EBV-related cancers (nasopharyngeal cancer) were similar in HIV-infected patients, and in organ recipients in this study [36]. Various researches presented co-carcinogenic influence of HPV and EBV on oncogenesis in oral cavity, oro- and nasopharynx, and larynx [32, 34, 35]. Among head and neck cancers, especially high incidence of oral and oropharyngeal cancers after solid organ transplantation was observed [5, 37].

It was reported that immunosuppressive agents influence various tumor-related signaling pathways thus affecting oncogenesis in different ways [30].

#### Head and neck cancers (HNC) potentially not related to viral infection

##### Lip cancer

Lip cancer (LC) is a neoplasm potentially not related to viral infection [36]. According to various authors, RTRs were more prone to develop LC after transplantation [33, 38, 39]. LC constituted 5–22.9% of all tumors in RTRs, and affected mainly male recipients [23, 31]. The risk of oncogenesis in the lip was higher in RTRs than in patients with CKD on hemodialysis [37]. Laprise et al. found that the risk of invasive LP in RTRs was 15-times higher than in heathy population [33]. According to Krynitz et al., RTRs presented 46-fold increased risk of LC [40].

The majority of LC cases in RTRs were invasive squamous cell carcinomas (SSCs) and were located on the vermilion of the lower lip [31, 33, 39]. It was suggested that the risk of LP occurrence in RTRs was related to the type and dosage of immunosuppressive drugs, as well as to the therapy timespan [31]. The longer and more aggressive treatment schedule was used, the higher the risk of lip oncogenesis was observed [33, 39]. A significant influence of immunodeficiency on lip oncogenesis was explained by achieving reduced, pre-transplantation risk of LP after immunosuppression discontinuation [39].

According to various authors, especially patients receiving cyclosporine A (CsA) were prone to develop LP [31, 39]. The risk of LP incidence in RTRs was also elevated by smoking and solar UV radiation [39]. Various authors concluded that immunosuppressive drugs used after organ transplant might have boosted UV-related changes in lip cells promoting carcinogenesis [33, 39].

##### Thyroid cancer

Thyroid cancer (TC) is another neoplasm that was suggested not to be related to viral infection [36]. A large cohort study on solid organ transplant recipients, conducted by Mowery et al. found increased risk of TC after transplantation [5]. RTRs constituted 50.5% of all participants in this study [5]. It was reported that the risk of TC was elevated both, after kidney transplantation and in patients with CKD on hemodialysis [41, 42].

The prevalence of TC was higher in patients with ESKD than in RTRs [41, 42]. It could have resulted from various metabolic changes induced by chronic kidney failure, mainly hypocalcaemia-induced secondary hyperparathyroidism and decreased serum levels of selenium [41].

##### Salivary gland cancer

Mowery et al. found that organ recipients were prone to develop salivary gland cancer (SGC) [5]. Piselli et al. reported the significantly increased risk of SGC after kidney transplantation [38]. Unfortunately, because of the lack of large cohort studies analyzing the prevalence of SGC in patients with CKD/RTRs, it could not be clearly stated weather these individuals are more prone to develop SGC than general population. Available literature on this subject is based mainly on case series and case reports.

#### Immunosuppressive agents used in patients with CKD

##### Biologic drugs (*lymphocyte-depleting antibodies, antithymocyte globulin, belatacept, rituximab, basiliximab, daclizumab, interleukin-2 receptor blockers*)

Biologic drugs, mainly lymphocyte-depleting antibodies, were considered to elevate the risk of cancers development, especially those related to viral infection [30]. The majority of studies emphasized the crucial role of EBV infection in oncogenesis induction [30].

##### Glucocorticosteroids

Glucocorticosteroids, typically used in combination with other immunosuppressive drugs, might promote carcinogenesis in direct and indirect ways [30]. A direct, pro-oncogenic activity is based on drug interference with lymphoid cells, whereas indirect activity is related to the increasing ability of cancer cells to escape from human immunosurveillance [30]. Glucocorticosteroids are able to deactivate lymphoid T and B cells and improve cancer cells resistivity to human immunity resulting in decreased neoplasm immunosurveillance [30]. The reports on the glucocorticosteroids-related carcinogenesis of head and neck in RTRs are lacking.

##### Anti-proliferative drugs (*azathioprine and mycophenolic a*cid)

Anti-proliferative drugs might increase the risk of oncogenesis via inducing mutagenic changes in DNA and leading to chronic oxidative stress [30]. Nevertheless, it was observed that mycophenolic acid (MA) decreased the risk of cancer incidence in RTRs, especially the risk of lymphoproliferative diseases [30]. It was found that RTRs receiving azathioprine developed significantly more aggressive head and neck cancers than patients given other immunosuppressive drugs, namely prednisone, cyclosporine, tacrolimus, mycophenolic acid, everolimus, and sirolimus [43].

##### Calcineurin inhibitors (CNIs) *(tacrolimus and cyclosporine)*

It was found that CNIs promoted tumor development and progression via increasing the expression of transforming growth factor β1 (TGF β1) and vascular endothelial growth factor (VEGF) both of which play a crucial role in oncogenesis [30]. CNIs also stimulated carcinogenesis via restraining anti-neoplastic immune response [30]. It was reported that long-term therapy based on CNIs might have played a major role in carcinogenesis of head and neck in organ recipients [43].

##### Mammalian target of rapamycin (mTOR) inhibitors *(everolimus and sirolimus)*

Phosphoinositide 3-kinase/protein kinase B/mammalian target of rapamycin (PI3K/AKT/mTOR) signaling pathway is a crucial controller of cellular growth and survival that is commonly upregulated in cancer [30]. It was suggested that mTOR inhibitors might reduce the risk of oncogenesis [30]. The antineoplastic activity of mTOR inhibitors is based on interfering with PI3K/AKT/mTOR signaling pathway via inhibiting various proteins that are involved in promoting tumor growth [30]. The well-known transmitting factors that are inhibited in this pathway are mainly vascular endothelial growth factor (VEGF) that is crucial for angiogenesis, epidermal growth factor (EGF), and insulin growth factor (IGF).

It was reported that patients receiving mTOR inhibitors after organ transplant had reduced risk of oncogenesis in comparison with those on alternative immunosuppressive therapies. According to that, mTOR inhibitors could constitute a group of drugs potentially reducing the frequency of post-transplant de novo cancers [38].

PI3K/AKT/mTOR pathway is frequently deregulated in HNSCC [38]. It was reported that PI3K/AKT/mTOR changes constituted approximately 80–90% of genomic alterations in HNSCC [38]. Studies on mTOR inhibitors implied that these agents could act as a sensitizer in combination with radiation therapy for HNSCC [38].

#### Treatment of head and neck post-transplant cancers

Studies presented that several post-transplant cancers (especially infectious-related tumors) could be treated by reducing the intensity of immunosuppression. Similarly, encouraging results were also obtained for infectious-related neoplasms after antiviral therapy in another study [41]. On the other hand, the risk of ESRD-related neoplasms, especially TC, was not reduced after immunosuppression reduction [41].

Interestingly, it was reported that incorporating therapy based on mTOR inhibitors instead of other immunosuppressive drugs in patients with non-melanoma skin cancers could have improved oncologic outcomes [30]. Nevertheless, the consensus on this subject has not been provided yet.

### Sinonasal dysfunctions

Nasal bleeding (epistaxis) is a common CKD-induced symptom [3]. Nasal cavity is one of the most common sites of bleeding in patients with uremia. In patients with CKD, epistaxis is mainly caused by the collection of toxic elements that in healthy people are eliminated by kidneys in urine. Other factors predisposing to nasal bleeding in CKD are anemia and coagulation dysfunctions [3]. It was suggested that urea was the most important cause of nasal bleeding [3]. Patients presenting epistaxis had very high level of blood urea (320 mg/100 cc) that was eliminated by nasal discharge [3]. In addition to that, epistaxis was exacerbated by bacteria that decompose urea to ammonia and colonize nasal cavity. It resulted in chemical rhinitis appearing as both, mucosal congestion and ulceration, and submucosal hemorrhages [3]. It was also implied that nasal bleeding resolved immediately after blood urea level was normalized, thus indicating the definitive role of urea in triggering epistaxis [3].

Other sinonasal manifestations, namely chronic and acute rhinosinusitis, invasive fungal rhinosinusitis or fungal ball are mainly observed in patients after organ transplant as a result of immunosuppression [44]. High prevalence of opportunistic infection in renal transplant recipients (RTRs) results from cytotoxic drugs and steroids incorporation, prolonged antibiotic therapy, drug-induced granulocytopenia as well as from metabolic abnormalities, namely uremia, hyperglycemia, and poor nutritional status [4]. Nevertheless, there is limited available data regarding rhinosinusitis in patients with CRD and in kidney recipients. Clear recommendations for the management of this disease especially in immunocompromised patients after renal transplantation are lacking.

A large-cohort study conducted by Ryu et al. revealed that the frequency and recurrence rate of rhinosinusitis did not increase after kidney transplantation [44]. Nevertheless, the authors suggested that patients with symptomatic rhinosinusitis should be appropriately treated surgically or pharmacologically before organ transplantation [44]. In accordance with the findings from this study, the authors concluded that sinonasal examination is not advised in asymptomatic individuals because no exacerbations were observed in renal transplant recipients [44]. Routine computed tomography of paranasal sinuses before organ transplant was also not recommended in asymptomatic individuals because of the high rate of false positive results [44].

The relatively low incidence of rhinosinusitis in RTRs might have resulted from persistent low-dose prednisone therapy withholding the inflammatory responses that frequently promote CRS [44].

It was stated that immunosuppression after organ transplantation is an independent risk factor for mucormycosis, an infection caused by *Mucoraceae* fungi. Additionally, renal failure also predisposed to this infection [45]. Sinonasal mucormycosis, the most frequent localization of this disorder in RTRs, usually presented as headache, facial swelling and pain especially over affected areas, nasal discharge, and necrotic lesions on the face, nasal cavities, or palates [45]. Godara et al. reported that the most common form of mucormycosis in RTRs was rhino-cerebral form with the prevalence reaching 56.25% [4]. The mortality rate of rhino-cerebral mucormycosis in this study was 33% [4]. Sun et al. also found that rhino-cerebral mucormycosis affected 57.1% of solid organ recipients, especially RTRs [45]. Rhino-cerebral mucormycosis was the most frequently observed form; nevertheless, there were also numerous cases of only sinus involvement [45]. Maxillary and ethmoid sinuses were mainly affected [45].

Maxillary sinus mucosal cyst (MSMC) is a benign and usually asymptomatic condition caused the obstruction of the duct of a seromucinous gland at sinus mucosa that leads to mucus collection and cystic dilatation of the affected gland [46]. It has not been established yet whether MSMCs are only a completely incidental finding, or whether they are an indicator of underlying sinus pathology [46].

According to Aydin et al., the incidence of MSMC did not differ between the cohort of organ recipients and general population [46]. Nevertheless, the authors found higher tendency of MSMC enlargement in RTRs than in healthy population [46]. They concluded that it could have been a result of immunosuppressive therapy and higher frequency of upper respiratory tract infections in organ recipients [46]. This observation should be analyzed with caution because of the lack of other studies on this subject.

Studies showed that the sense of smell could be affected in patients with CKD. It was reported that patients with CKD, mainly those with ESDK on dialysis, had moderately but significantly decreased olfactory function [47, 48]. The prevalence of olfactory loss reached 56% of patients with ESRD [48]. While olfactory identification and discrimination remain under control of higher cognitive aspects of olfactory processing, olfactory threshold is controlled by peripheral olfactory pathways [47].

In the study conducted by Landis et al. olfactory identification and discrimination were mainly affected in patients with CKD while thresholds seemed to remain unchanged [47]. This observation indicated that both, peripheral and central nervous changes led to this olfaction disorder [47]. It was also reported that odor identification was significantly reduced in the majority of subjects with CKD (approximately 70%) and ESRD (approximately 90%) [48]. Olfactory thresholds that express the minimal intensity of odor to be detected by an individual were higher only in patients with ESRD, suggesting that olfactory correlated with the severity of renal disease [48]. Interestingly, olfactory dysfunction in CKD seemed to be reversible [47]. This finding was based on the observation of improvement of proper olfaction after renal transplantation, as well as after dialysis session [47]. The exact blood markers that could have affected olfactory function remain unknown. However, olfactory function could be a marker of uremia-related neurologic dysfunction [47]. It was speculated that urea could be responsible for olfactory impairment because of its negative effect on both, peripheral nerve conduction and cognitive functions, nevertheless the results obtained by various authors were inconsistent [47].

### Voice changes

Voice changes were commonly observed in patients with ESKD and resulted mainly from the ESKD-induced influence on the respiratory and phonatory systems [49, 50]. Patients with ESKD may experience voice disorders mostly because of ESKD-triggered excessive fluid and toxins accumulation, and acid–base imbalance [50].

Patients with ESKD commonly presented vocal cord edema, decreased pulmonary function or abnormal coordination between central nervous system and peripheral phonatory structures, all of which subsequently led to voice changes [50]. Individuals with ESKD expressed a clinical evidence of voice abnormalities acoustically and aerodynamically [49]. Hoarseness was detected in 24–60% of subjects with ESKD after each hemodialysis session and usually lasted for few hours after hemodialysis completion [50]. It was found that patients with ESKD on hemodialysis might have suffered from temporary post-dialysis hoarseness as a result of hemodialysis-induced dehydration, reduction of the vocal cord size and increase in subglottic pressure [50]. Precise analysis of acoustic parameters conducted by Jung et al. revealed increase in both, fundamental frequency (F0) and habitual pitch (HP), and decrease in noise-to-harmonics ratio (NHR) and maximal phonation time (MPT) after hemodialysis [50]. Hassan et al. also presented increased F0 and reduced MPT in patients with CKD [49]. In contrast to Jung et al. finding, in the study conducted by Hassan, NHR was elevated [49, 50]. The authors suggested that it could have emerged from the lack of phonation control leading to improper glottis opening [49, 50].

Besides the fact that, a number of patients with ESKD experience deterioration of voice quality after hemodialysis session during which they lost more weight than expected, the association between weight loss and hoarseness in this group was not confirmed in clinical studies [50]. This observation could have emerged from the fact that weight reduction might not be a proper value expressing the reduction of vocal cord size [50]. Additionally, voice changes might have resulted from disturbances in other phonation-related factors, namely laryngeal muscles, subglottic pressure, vocal tract or pulmonary function that interfere with the linear association between weight fluctuation and voice changes [50]. Deterioration of voice quality might also have been influenced by hemodialysis-induced fatigue [50].

There were also rare cases of CKD-induced hypocalcemia-related laryngospasms that appeared as a result of enhanced reflex excitability of the recurrent laryngeal nerves at the neuromuscular junctions. Nevertheless, this topic has not been precisely discussed in patients with CKD yet.

### Deep neck infections (DNIs)

It was implied that CKD predisposes to DNIs, a polymicrobial, rapidly progressing and life-threatening disease [51].

DNIs spread in deep cervical spaces that are formed by fascia [52]. Clinical presentation of these infections depends on the site of infection origin, infection extension and the pressure effects of edema or accumulated fluid on surrounding tissues [52]. Depending on the site of infection origin, patients may suffer from sore throat, trismus, dental pain, dyspnea, stridor, dysphagia, odynophagia, or neck asymmetry with tenderness, swelling and erythema [52]. Fever and toxic condition are commonly observed [52]. DNIs require aggressive and immediate treatment because of the life-threatening nature of the disease [52]. The prognosis of successful DNI treatment in patients with CKD, especially in RTRs, is worse than in general population [51]. Reoperations and longer hospitalizations are often required in these patients [53].

Studies presented that individuals with CKD, especially those on dialysis, were more prone to develop DNIs [51, 54–56]. Additionally, CKD predisposed to the extension of DNI into the mediastinal space [57]. In general, subjects with CKD expressed approximately three-times increased risk of serious infection incidence, while the need for hospitalization because of serious inflammation was almost ten-times greater in dialysis patients than in general population [54]. Nevertheless, large cohort studies analyzing the precise correlation between CKD and DNIs are lacking.

According to studies, the most important factor predisposing to DNIs in CKD population is uremia, however, this hypothesis lacks strong evidence based on large cohort studies [51, 54].

Uremia interferes with primary host defense mechanisms subsequently elevating the risk of bacterial infections [54]. Uremia, commonly accompanied by impaired glucose metabolism, secondary hyperparathyroidism, iron accumulation, malnutrition and dialysis, leads to neutrophil dysfunction in patients with CKD and RTRs [54]. Dysfunctional neutrophils express malfunctioning chemotaxis, degranulation and phagocytosis, subsequently failing to prevent CKD host from developing infection [54]. A crucial role in developing DNIs in RTRs is additionally played by constant immunosuppression and the immunity alterations that favor the growth of opportunistic organisms [56].

According to Chang et al. the risk of developing DNI in population with ESKD is two-times elevated [51]. In the study conducted by Yang et al., CKD constituted the third most common condition predisposing to DNIs following DM and nasopharyngeal cancer after radiotherapy [58]. Motahari et al. observed that ESKD was defined as a condition precipitating DNIs in 3.1% of all DNIs cases [59].

## Conclusion

Otorhinolaryngological abnormalities are not rare complications of CKD and its treatment. Patients with CKD are prone to develop mainly sensorineural hearing loss, tinnitus, vestibular dysfunction, recurrent epistaxis, opportunistic infections including oropharyngeal candidiasis or rhino-cerebral mucormycosis, taste and smell changes, deep neck infections, phonatory dysfunction, mucosal abnormalities, gingival hyperplasia, halitosis or xerostomia. Individuals on immunosuppressive therapy after kidney transplantation present increased risk of carcinogenesis, both related and not-related to latent viral infection. The most commonly viral-related neoplasms observed in these patients are oral and oropharyngeal cancers, whereas the majority of not-related to viral infection tumors constitute lip and thyroid cancers.

CKD-related otorhinolaryngological dysfunctions are often permanent, difficult to control, have a significant negative influence on the patient’s quality of life and can be life threatening. Because of the high prevalence of otorhinolaryngological complications induced by CKD itself and its treatment we concluded that patients with CKD, including those after organ transplantation, require frequent and long-term examination conducted by an experienced otorhinolaryngologist. It is especially crucial because some of these complications could be reversible when early detected and managed. In addition to that, the progression of some of these dysfunctions could be inhibited by introducing proper treatment, and some could be improved after treatment modification.

The predisposition to several otorhinolaryngological complications in patients with CKD, and the relationship between them and CKD was widely explained, whereas the correlation between the rest of them and CKD remains unclear. It mainly results from the lack of large cohort studies and conflicting results of the existing ones. Accordingly, further studies on this subject are necessary.
